# Fungal-Host Interaction: Curcumin Modulates Proteolytic Enzyme Activity of *Candida albicans* and Inflammatory Host Response *In Vitro*

**DOI:** 10.1155/2018/2393146

**Published:** 2018-08-15

**Authors:** Emily Chen, Bruna Benso, Dalia Seleem, Luiz Eduardo Nunes Ferreira, Silvana Pasetto, Vanessa Pardi, Ramiro Mendonça Murata

**Affiliations:** ^1^Herman Ostrow School of Dentistry, University of Southern California, Los Angeles, CA, USA; ^2^School of Dentistry, Pontificia Universidad Católica de Chile, Santiago, Chile; ^3^College of Dental Medicine, Western University of Health Sciences, Pomona, CA, USA; ^4^School of Dental Medicine, East Carolina University, Greenville, NC, USA; ^5^Brody School of Medicine, East Carolina University, Greenville, NC, USA

## Abstract

Current treatments for *Candida albicans* infection are limited due to the limited number of antifungal drugs available and the increase in antifungal resistance. Curcumin is used as a spice, food preservative, flavoring, and coloring agent that has been shown to have many pharmacological activities. Thus, this study evaluated the modulatory effects of curcumin on major virulence factors associated with the pathogenicity of *C. albicans*. The minimum inhibitory concentration (MIC) of curcumin against *C. albicans* (SC5314) was determined. Biofilm formation was quantified and the proteinase and phospholipase secretion was measured. The cytotoxicity was tested in oral fibroblast cells. A cocultured model was used to analyze the gene expression of proinflammatory cytokines (IL-1*β*, IL-1*α*, and IL-6) from host cells, as well SAP-1 and PLB-1 by RT-PCR. The MIC was between 6.25 and 12.5 *µ*M, and the activity of proteinase enzyme was significantly decreased in biofilms treated with curcumin. However, proteinase gene expression was not downregulated after curcumin treatment. Furthermore, gene expressions of host inflammatory response, IL-1*β* and IL-1*α*, were significantly downregulated after exposure to curcumin. In conclusion, curcumin exhibited antifungal activity against *C. albicans* and modulated the proteolytic enzyme activities without downregulating the gene expression. In host inflammatory response, curcumin downregulated IL-1*β* and IL-1*α* gene expression.

## 1. Introduction

*Candida albicans* is a prevalent opportunistic fungus that becomes pathogenic in patients with reduced immune competence or in individuals with an imbalance of competing bacterial microflora [1–3]. The pathogenicity of the *Candida* species is attributed to critical virulence factors, such as the ability to evade host defenses, adhere to surfaces (on tissues and medical devices), biofilm formation, and the production of proteolytic enzymes, such as secreted aspartyl proteases (SAP) and phospholipases [4].

Current treatments for *C. albicans* infection consist of topical and systemic pharmaceutical antifungal agents [5]. Antifungal resistance has been increasing due to the limited number of antifungal treatments available and the widespread use of these drugs [6, 7]. Therefore, the discovery of new and effective antifungal therapeutic agents is a necessity. Natural compounds are readily available in many foods and beverages. They are a source of molecules with antimicrobial, anti-inflammatory, and antioxidant potential [8].

Polyphenols are secondary metabolites found in many plants, which have been used for thousands years in traditional herbal remedies due to their diverse biological activities [9]. Protective effects of such flavonoids have been reported against cancer, cardiovascular diseases, diabetes, infectious disease, as well as age-linked conditions, which renders them potential therapeutic agents [10]. Curcumin is a yellow pigment derived from the roots of *Curcuma longa* plants that is commonly used as a spice, food preservative, flavoring, and coloring agent in Asia and India [10–12]. Curcumin has been shown to have many pharmacological activities including antioxidant, anti-inflammatory, antiviral, antitumor, and antibacterial activities [13]. Moreover, curcumin acts as a photosensitizer for photodynamic therapy with clinical application for pharyngotonsillitis, with the proposal to reduce the use of antibiotics [14].

Based on the indexed literature, we hypothesized that curcumin can affect the virulence factors of *Candida albicans* and the host immune response to the pathogen. The aim of this study was to investigate the modulatory effects of curcumin *in vitro* in some virulence factors associated with the pathogenicity of *Candida albicans*. Proteolytic enzyme activities secreted by *C. albicans* were quantified in addition to gene expression of inflammatory cytokines marker of the host in a coculture system. Ultimately, this study explored the mechanisms by which curcumin can modulate the pathogenicity of *Candida albicans* and validated the pharmacological effects of curcumin.

## 2. Materials and Methods

### 2.1. Susceptibility Test

Antimicrobial activity of curcumin (Sigma-Aldrich; St. Louis, MO) was tested *in vitro* according to the NCCLS guidelines against *Candida albicans* strain (ATCC SC5314/MYA2876). Curcumin concentrations ranged from 1.5 to 400 *μ*M. Fluconazole (322 *μ*M) (Sigma) and 1% dimethyl sulfoxide (DMSO) (v/v) (Sigma-Aldrich; St. Louis, MO) served as a positive control and vehicle control, respectively. The minimum inhibitory concentration (MIC) was determined using an inoculum of 5 × 10^3^ CFU/ml. *C. albicans* were grown in RPMI-1640 (Lonza, Walkersville, MD) in a 96-well plate, and incubated for 24 h at 37°C in 5% CO_2_. After 24 h, the MIC was determined visually, and the minimum fungicidal concentration (MFC) was found by subculturing 20 *μ*l from each concentration above the MIC on Sabouraud dextrose agar (Becton Dickinson, Franklin Lakes, NJ) for 48 hours at 37°C in 5% CO_2_ [15].

### 2.2. Biofilm Assay

One milliliter of 1 × 10^6^ CFU/ml *C. albicans* inoculum was added in each well of a sterile 24-well plate, suspended in yeast nitrogen base medium (Becton Dickinson, Franklin Lakes, NJ) with 50 mM of glucose. The plate was incubated for 24 h (37°C in 5% CO_2_) to allow initial biofilm growth and adhesion to the plate surface. Biofilms were then treated every 24 h using curcumin concentrations of 62.5 *μ*M and 125 *μ*M (10x MIC and 20x MIC resp.) for three days. Before each treatment, biofilms were washed with 500 *μ*l of PBS and replenished with 900 *μ*l of fresh medium and 100 *μ*l of curcumin treatments. The 1% ethanol was used as vehicle control, and fluconazole (1 mg/ml) served as a positive control. On the fifth day, biofilms were washed with PBS and the biomass was measured.

PBS (1 mL) was added to each well, and the biofilm was suspended to disrupt the biofilm formation. Viability and colony formation unit (CFU) of *C. albicans* were determined by plating 20 *μ*l of the suspended biofilm solution on Sabouraud dextrose agar plates (Becton Dickinson, Franklin Lakes, NJ). The plates were incubated for 24 h at 37°C in 5% CO_2_, and the number of *C. albicans* colonies was counted. To determine the dry weight of the biofilm sample, *C. albicans* suspended in PBS solution was centrifuged at 10,000 rpm for 5 minutes. The supernatant was discarded and the sample was placed in a speed vacuum to dry for 40 minutes, and dry biofilm mass was determined [16].

### 2.3. Cell Viability Test

Oral fibroblast cells (ATCC: CRL2014) were cultured in Dulbecco's modified Eagle's medium (DMEM) (Lonza, Walkersville, MD) with 10% fetal bovine serum (FBS, Lonza, Walkersville, MD) at 37°C in 5% CO_2_. Fibroblast cells (1 × 10^5^ cells/ml) were first seeded in each well of a 96-well plate in DMEM with 10% FBS, and the plates were incubated for 24 h at 37°C in 5% CO_2_. Then, cells were treated with curcumin (1.5–640 *μ*M), and the plates were incubated at 37°C in 5% CO_2_ for 24 h. Cell viability was measured by the fluorometric method (Cell Titer Blue, Promega Corp, Madison, WI) in a SpectraMax M5 microplate reader (Molecular Devices Sunnyvale, CA) with 550 nm (Ex)/585 nm (Em) wavelength [17].

### 2.4. Proteinase and Phospholipase Enzyme Secretion Assay

Proteinase and phospholipase enzyme secretion assays were conducted as previously performed by Santana et al. [15]. Biofilms of *C. albicans* were grown as described before and treated for 72 h using curcumin (62.5 *μ*M and 125 *μ*M) and the standards: phospholipase A2 (Sigma-Aldrich; St. Louis, MO) and trypsin (Lonza, Walkersville, MD) for proteinase assay. The vehicle control was 1% ethanol. *C. albicans* biofilms were sonicated, and the proteinase enzyme activity was determined by mixing the supernatant of the biofilm solution with 1% azocasein at 1 : 9 (v/v) for 1 h at 37°C in 5% CO_2_. Then, 500 *μ*l of 10% trichloroacetic acid was added to stop the reaction. The solutions were centrifuged for 5 minutes at 10,000 rpm. The supernatant (500 *μ*l) was combined with 500 *μ*l of NaOH and incubated at 37°C in 5% CO_2_ for 15 min. The absorbance was read at 440 nm using a spectrophotometer [5, 13, 17]. The phospholipase enzyme activity is determined by mixing the supernatant of the biofilm solution with phosphatidylcholine substrate for 1 h at 37 °C in 5% CO_2_. Absorbance was read in a spectrophotometer at 630 nm [13, 15, 18].

### 2.5. Coculture Model Quantitative Real-Time PCR

Fibroblast cells (1 × 10^5^ cells/ml) were seeded in a 96-well plate in DMEM medium with 10% FBS and incubated at 37°C in 5% CO_2_ for 24 h. The medium was replaced, and *C. albicans* inoculum of 5 × 10^3^ to 2.5 × 10^3^ CFU/ml in DMEM without FBS was added. Fibroblast cells and *C. albicans* were treated with 20 *μ*M and 40 *μ*M (subcytotoxic dose) of curcumin. The plates were incubated for 24 h. The vehicle control tested was 1% ethanol, while fluconazole was the positive control. Total RNA was isolated from fibroblast cells and *C. albicans*. The RNA was purified using the RNeasy MiniKit (Qiagen, Valencia CA) and the RiboPure Yeast Kit (Life Technologies, Carlbad, CA), respectively. A NanoPhotometer P360 (Implen; Westlake Village, CA) was used to quantify the total RNA extracted. Reverse transcription of the RNA into cDNA was carried out using iScript Advanced cDNA synthesis Kit for RT-qPCR (BioRad, Hercules, CA) according to the manufacturer's instructions. Real-time PCR was conducted using iQ SYBR Green Supermix (BioRad, Hercules, CA). The *C. albicans* primers for the genes secreted aspartyl proteinases-1 (SAP-1), phospholipase B-1 (PLB-1), and ACT-1 (housekeeping) at 10 *μ*M were used [19]. Based on previous analysis using the RT^2^ Profiler PCR Array Kit (Qiagen, Valencia CA), the following fibroblast genes were selected: IL1-*α* (Qiagen Gene ID#: 3552), IL1-*β* (Qiagen Gene ID#: 3553), IL-6 (Qiagen Gene ID#: 3569), and GADPH (Qiagen Gene ID#: 2597). PCR amplification was performed using 20 *μ*l of the reaction mix in each of the 96-well plate. The reactions were conducted at 95°C for 3 minutes, followed by 40 cycles of 15 seconds at 95°C and 1 minute at 60°C. After PCR, the melting curve was obtained by incubating the samples at increasing increments of 0.5°C from 55°C to 95°C.

### 2.6. Statistical Analysis

Data were tested for normal distribution by Shapiro–Wilks' test, and the equivalence of variances were tested by Levene's test. All data were expressed as the mean ± SEM using one-way analysis of variance (ANOVA) and Dunnett's multiple comparison tests in relation to the vehicle. The level of statistical significance was set at 0.05. The lethal dosage (LD_50_) was found using nonlinear regression analysis by MasterPlex 2010 Reader Fit. PCR analysis was performed using the ΔΔCt method.

## 3. Results

**Figure 1:**
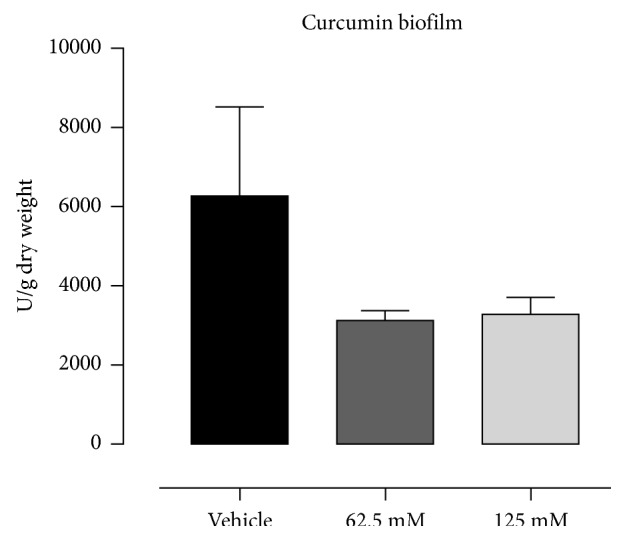
Mean and SD of *Candida albicans* biofilm expressed in CFU/grams of dry weight after treatment with curcumin.

The MIC for curcumin against *C. albicans* was in a range between 6.25 *μ*M and 12.5 *μ*M. The biofilm assay results showed a decrease in the mass of biofilms treated with curcumin (62.5 *μ*M and 12.5 *μ*M) in relation to the vehicle control (Figure 1). However, the results were not statistically significant (*p* > 0.05). Concentrations of curcumin below 40 *μ*M showed no significant cytotoxicity against oral fibroblast cells when compared to the vehicle (data not shown), and the LD_50_ was 48.75 *μ*M.

**Figure 2:**
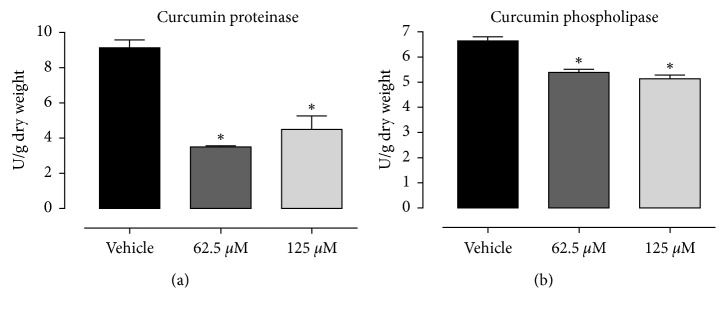
Mean and SD of *Candida albicans* proteinase (a) and phospholipase (b) enzyme secretion expressed in U/grams of dry weight after treatment with curcumin (62.5 *μ*M and 125 *μ*M). ^*∗*^Statistical difference in relation to vehicle control, *p* < 0.05, ANOVA, Dunnett's.

**Figure 3:**
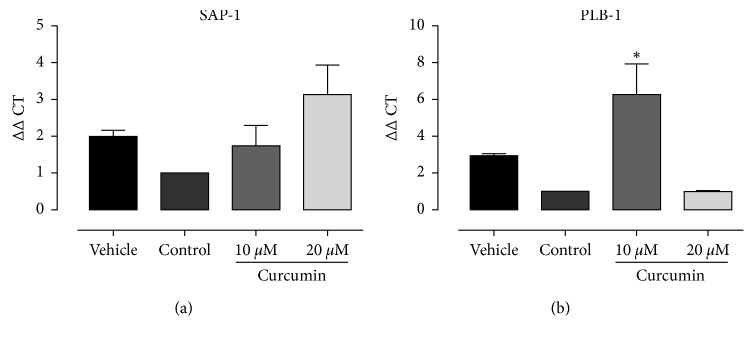
Real-time quantitative information about gene expression of SAP-1 (a) and PLB-1 (b) after curcumin treatments (10 *μ*M and 20 *μ*M) in oral fibroblast cells infected by *C. albicans*. ^*∗*^Statistical difference between curcumin treatments and control in comparison with vehicle, *p* < 0.05 ANOVA, Dunnett's.

**Figure 4:**
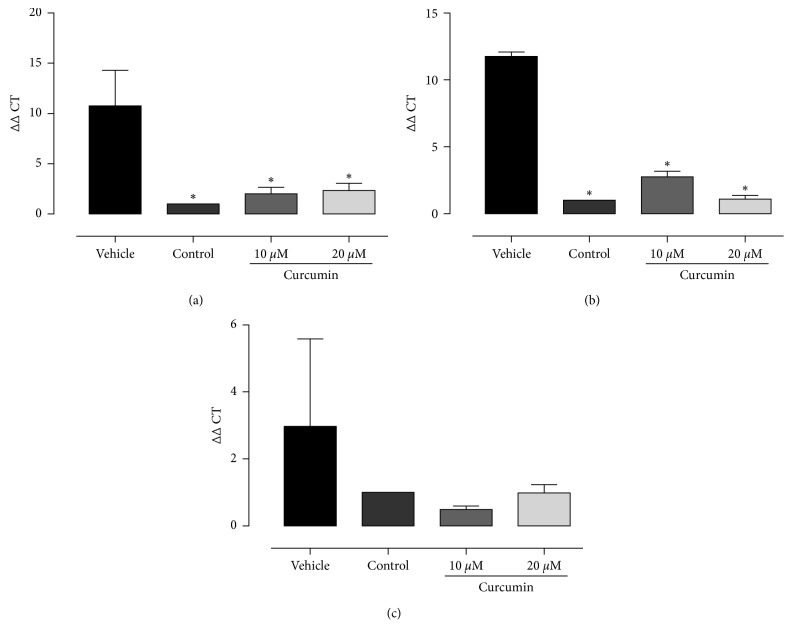
Real-time quantitative information about gene expression of IL-1*α* (a), IL-1*β* (b), and IL-6 (c). ^*∗*^Statistical difference between curcumin treatments and control in comparison with vehicle, *p* < 0.05, ANOVA, Dunnett's.

After treatments with curcumin at 62.5 *μ*M and 125 *μ*M, there was a significant decrease (*p* < 0.05) in the proteinase and phospholipase enzyme activity when compared to the vehicle (Figures 2(a) and 2(b)). There were no differences in the expression of SAP-1 after exposure to curcumin (Figure 3(a)). The treatment with 10 *μ*M curcumin significantly increased the PLB-1 gene expression in comparison to the vehicle. However, there was no difference between curcumin at 20 *μ*M and vehicle (Figure 3(b)). The expression of host inflammatory markers showed a significant downregulation in IL-*α* and IL1-*β* with curcumin treatment at 10 *μ*M and 20 *μ*M. There were no changes in the expression of IL-6 gene for both curcumin treatments (Figure 4).

## 4. Discussion

The resistance of *Candida* species to conventional antifungal agents, such as triazoles, represents a major challenge for the treatment of candidiasis especially in individuals with diminished immune response, for example, in HIV patients. Natural compounds are potential therapeutic agents that may be considered for the treatment of fungal infection because of their antimicrobial benefits. Over the past 30 years, the FDA has recognized 69% of 109 small molecules from natural products or derivates as having antimicrobial effects [20].

Curcumin stands as a potential antimicrobial natural compound, which is incorporated as an important traditional remedy spice used by the traditional Asian and Indian culture. However, scientific validation of its antimicrobial efficacy, toxicity effects, and mechanism of action are necessary to establish its safety for therapeutic purposes. Thus, this study demonstrated the curcumin effects on virulence factors of *C. albicans*, including the analysis of gene expression.

Curcumin has been reported to have antifungal activity against various strains of *Candida*, including *Candida albicans* (ATCC 10261), with a minimum inhibitory concentration (MIC) ranging from 250 to 2000 *µ*g/ml (0.68 mM to 5.4 mM) [11, 21]. In this study, we used *C. albicans* SC5314 strain, and the MIC was found in the range of 6.25–12.5 *µ*M. *C. albicans* 5314 was used because the genome is fully described, with well-known molecular patterns and phenotypes. In addition, the biofilm formation by this strain is well established in several studies [22, 23].

In the biofilm assay, ten times of MIC concentration (62.5 *µ*M and 125 *µ*M) were tested because biofilms have a denser network of yeasts and hyphal population that are more resistant to drug diffusion than to planktonic counterparts. It was found that both concentrations of curcumin did not significantly reduce the colony formation in the biofilms normalized by the dry weight of the samples. Possible explanation for the lack of the significant CFU reduction/dry weight is that curcumin did not drastically affect the composition of biofilms. However, this hypothesis needs to be further investigated by studying the polysaccharides and protein composition of the biofilm samples upon treatment with the compound. Similarly, the biofilm's dry weight (data not shown) did not show significant differences among the groups, signifying that the total biomass compositions of all fungal cells, including dead/live cells, were not affected with treatment of any compound.

The lethal dosage or 50% cell viability (LD_50_) was 48.75 *μ*M, which is important to ensure the therapeutic safety level when considering *in vivo* studies as well as human clinical trials. It should also be noted that in coculture models, curcumin has more sensitive and profound effect on the morphology and distribution of fibroblast cells, as this model represents “naked-cells,” that have a more susceptible cell response than cells tested under clinically relevant conditions.

Proteinases and phospholipases are enzymes secreted by *Candida albicans* often associated with tissue degradation, hyphal formation, and host invasion, which are critical factors linked to the pathogenicity of *C. albicans* [24, 25]. Proteinase and phospholipase enzyme activities were reduced using curcumin at 62.5 *μ*M and 125 *μ*M. These results suggest that one possible curcumin mechanism of action involves inhibition of proteinase secretion, which is an important virulence factor [21]. This finding is consistent with the results reported by Neelofar et al. [11], in which curcumin decreased proteinase secretion by 53% in *C. albicans* compared to the vehicle control group.

SAP proteins are often associated with virulence factors able to elicit a destructive effect on the host tissue during mucosal infections, as they facilitate hyphal invasion and activate the degradation of E-cadherin, a major protein present in epithelial cell junction [24, 25]. In this current study, the effect of curcumin on SAP-1 gene expression was analyzed.

There was no significant downregulation in SAP-1 gene expression after treatment curcumin at 10 *µ*M and 20 *µ*M (Figure 3(a)). One possible explanation is based on a negative feedback mechanism modulating gene expression. Gene expression of proteases may play an important role in regulating the enzyme activity of proteases. Thus, as indicated by the significant decrease in proteolytic enzyme activities of phospholipases and proteinases, there may be a negative feedback inhibition regulating their respective gene expression. However, this hypothesis needs to be confirmed through further molecular studies.

The ability of *C. albicans* to attach to the host tissue is considered a key pathogenic characteristic and an important virulence factor. Phospholipase B (PLB) proteins were shown to have hydrolytic activity, as they hydrolyze acyl ester bonds in phospholipids and lysophospholipids and catalyze lysophospholipase-transacylase reactions. PLB multigene family encodes for CaPLB5, a putative secretory protein with a predicted GPI-anchor attachment site [26]. The PLB-1 gene expression after curcumin treatment was also evaluated. Although curcumin in lowest concentration increases the expression of PLB-1, this was not reflected in the enzymatic activity.

Host immune defense plays a critical antagonistic role during fungal infections, where the pathogenic state of candidiasis is marked by an increase in the proinflammatory cytokines [27, 28]. Gingival fibroblasts are major actors in the host immune defense against *C. albicans* infection. Fibroblasts express dectin-1 on the cell surface that recognizes *C. albicans* and active the inflammatory response by secreting inflammatory cytokines, such as IL-1*β*, IL-1*α*, IL-6, and IL-8 [29, 30]. Proinflammatory cytokines play an important role in the pathogenesis of many inflammatory diseases [31]. In this study, the gene expressions of the proinflammatory cytokines, IL1-*α*, IL1-*β*, and IL-6, were analyzed using host oral fibroblast cells infected with *C. albicans* in a coculture model.

We demonstrated that curcumin can reduce the IL-1*α* and IL-1*β* gene expression of fibroblasts exposed to *C. albicans* infection. Similar results were observed in *C. albicans* treated with monolaurin [32]. The anti-inflammatory property of curcumin is well established and has been demonstrated in different cells [33, 34]. In agreement with our results, curcumin has been reported to block the release of IL-1 in bone marrow stromal cells, colonic epithelial cells, and human articular chondrocytes [35].

These cytokines promote the inflammation by the activation of innate immune response and the induction of cyclooxygenase type 2. Furthermore, these cytokines also increase the expression of adhesion molecules, synthesis of nitric oxide, and the release of other cytokines [36]. However, IL-6 gene expression was not affected after curcumin treatments. In some systemic diseases, the IL-1 blockade reduces the levels of IL-6 [36]. Thus, IL-6 gene expression appears to be more associated with IL-1*α* and IL-1*β* levels than with the effects of curcumin.

Curcumin suppressed the production of inflammatory cytokines via regulation of molecular targets and transcription factors [37]. In vascular smooth muscle cells, curcumin inhibits LPS-induced inflammation by suppressing the activation of TLR4, inhibiting phosphorylation of ERK1/2 and p38 MAPK, preventing nuclear translocation of NF-κB, and reducing NADPH-mediated intracellular ROS production [38].

Although gingival fibroblasts are nonprofessional immune cells, they also express other pattern recognition receptors, such as TLRs, that recognizes *C. albicans* molecular patterns [39]. Activation of TLRs leads to activation of transcription factors such as NF-κB and interferon regulatory factors that induces the expression of various proinflammatory cytokines [40]. The downregulation of IL-1*α* and IL-1*β* induced by curcumin during the exposure to *C. albicans* could be related to the inhibition of TLR-MAPK/NF-κB pathways. Others anti-inflammatory mechanisms of curcumin in the *C. albicans* infection should be elucidated.

## 5. Conclusion

Curcumin had a slight antifungal activity against *Candida albicans* (SC5314). Curcumin reduces the proteolytic enzyme activities of phospholipases and SAPs without downregulating the gene expression. Furthermore, curcumin can modulate the host inflammatory response by decreasing gene expression of IL-1*β* and IL-1*α*. Future direction for research may involve studying the synergistic effects of curcumin and other conventional therapies on biofilm models. In addition, the curcumin efficacy in *C. albicans* strains isolated from clinical samples with different virulence profiles should be tested.

## Data Availability

The data used to support the findings of this study are available from the corresponding author upon request.
